# What Is? (Poetry)

**Published:** 1881-05

**Authors:** Ella Wheeler


### PROOF OF ANIMAL LIFE IN OTHER PLANETS.

[From the London Telegraph.]

Two interesting problems which have long perplexed the scientific world appear to have been at last definitely solved by the eminent geologist Dr. Hahn. These questions are, first, whether or not celestial bodies, other than the earth, belonging to our solar system, are inhabited by animate beings, and secondly, whether the meteoric stones from time to time cast upon the surface of this globe emanate from incandescent comets or from volcanic planets. That they at no time formed a part of the earth itself has been conclusively demonstrated.

Dr. Hahn has recently completed a series of investigations upon some of the huge meteoric stones that fell from the skies in Hungary during the summer of 1866. Thin laminæ of these mysterious bodies, subjected to examination under a powerful microscope, have been found to contain coralline and spongeous formations, and to reveal unmistakable traces of the lower forms of vegetation. All the organisms, animal and vegetable, discovered by Dr. Hahn in the delicate stone shavings he has thus dealt with, indicate the condition of their parent world to be one of what is technically termed "primary formation." But the presence of water in that world is proved by the fact that the tiny petrified creatures revealed by the magic of the lens one and all belong to the so-called subaqueous classes of animals. They could not have existed in comets, at least if the assumption be correct that these are in a state of active combustion.

---

Sunday School teacher (reprovingly)—"Boys, do you know what day this is?" Street boy—"Hi, fellers! here's a cove don't know what day this is! I guess he's been out all night!"

---

### WHAT IS?

Wealth and glory and place and power—
What are they worth to me or you?
For the lease of life runs out in an hour,
And death stands ready to claim his due.
Sounding honors, or heaps of gold—
What are they all when all is told?

A pain or a pleasure, a smile or a tear—
What does it matter which we claim?
For we step from the cradle into the bier,
And a careless world goes on the same.
Hours of gladness, or hours of sorrow—
What will it matter to us to-morrow?

Troth of love, or vow of friend,
Tender caresses, or cruel sneers—
What do they matter to us in the end?
For the brief day dies, and the long night nears.
Passionate kisses, or tears of gall—
The grave will open and cover them all.

Homeless vagrant, or honored guest,
Poor and humble, or rich and great,
All are racked with the world's unrest,
All must meet with the common fate.
Life from childhood till we are old—
What is all when all is told?

—*Ella Wheeler.*

### FARRAGUT IN MOBILE BAY.

[From an "An August Morning with Farragut," by E. C. Kinney, in the June "Scribner," which also contains a description of the new Farragut statue, with illustrations.]

"Happily for the fleet and for the country, there was a man in command that day equal to the emergency—a man whose eagle eye grasped every detail of the fight, while he possessed the skill to direct and the nerve and ability to execute. There was no time for doubt or delay. Had he hesitated, the fortune of the day must have been against us. The Admiral was standing in the futtock shrouds, under the main-top—a position above the smoke, from which he could take in the whole situation, and could communicate with the pilot in the main-top, and with the fleet-captain and executive officer on the deck beneath. For several years there has been a discussion in the papers and magazines of the country as to the Admiral's being 'lashed to the rigging.' The writer has no light to throw on the subject. Farragut was standing in the shrouds, as described, when the writer went on deck, and he remained there until the Hartford had passed beyond the range of the fort; but there were not more than two or three persons on board who knew anything about his being fastened in place. The first heard of it in the fleet was some three or four weeks after the fight, when the New York papers were received. Various rumors have been circulated as to the fact, one of which was that the Admiral took a rope's-end with him when he went aloft, and secured it so as to prevent his falling on deck in case of accident. This is the story which was current on shipboard at that time, and was generally believed. Since the incident has been under discussion in the papers the 'real facts' in the case have been made known, and will stand in history on the unquestioned authority of Fleet-Captain Drayton and of Flag-Lieutenant J. Crittenden Watson, of the Admiral's staff. This is to the effect that Captain Drayton, seeing the Admiral in the rigging, and fearing he might be killed by a fall on deck in case he were wounded, ordered an old quartermaster to take a rope's-end and secure it around him, so that he would be prevented from falling. The writer is disposed to believe that the Admiral was so absorbed in watching the fight that he did not know at the moment the precautions taken for his safety by his fleet-captain. But whatever doubt may attach to this particular incident—of which so much has since been made, while so little was thought of it at the time—there is no chance for doubt as to the Admiral's action. Finding that the Brooklyn did not start ahead, he hurriedly inquired of pilot Freeman, in the main-top, if there was depth enough for the Hartford to pass to the left of the vessels in front. Receiving an affirmative reply, he said, 'I will take the lead,' and immediately ordered the ship 'ahead fast.'

"On board a war steamer the engines are directed by the tap of a bell, the wires connected with which lead to the quarter-deck. One stroke of the bell means 'go ahead;' two, 'stop'; three, 'back'; and four, 'go ahead as fast as possible.' Leaning down through the shrouds to the officer on deck at the bell-pull, the Admiral shouted, 'Four bells, *eight bells*, SIXTEEN BELLS! Give her all the steam you've got.' The order was instantly transmitted, and the old ship seemed imbued with the Admiral's spirit, and, running past the *Brooklyn* and the monitors, regardless of fort, ram, gun-boats, and the unseen foe beneath, dashed ahead, all alone, save for her gallant consort, the *Metacomet.*"

---

HENRY BERGH, President of the New York Society for the Prevention of Cruelty to Animals, advocates flogging in every State of the Union, as well as by the national government, in place of imprisonment. He says: "There are twelve thousand criminals in our State prisons, and eighty thousand more in our jails and our penitentiaries, of whom New York City alone supplies fifty-five thousand. The criminal classes cost $6,000,000 a year in New York, and there is one church to every two thousand citizens, and a rum-shop to every seventy-five."

---

### AUF WIEDERSEHEN.

IN MEMORY OF J. T. F.

[From the Atlantic Monthly.]

Until we meet again! That is the meaning
Of the familiar words that men repeat
At parting in the street.
Ah, yes, till then! but when death intervening
Rends us asunder, with what ceaseless pain
We wait for the Again.

The friends who leave us do not feel the sorrow
Of parting as we feel it who must stay
Lamenting day by day,
And knowing, when we wake upon the morrow,
We shall not find in its accustomed place
The one beloved face.

It were a double grief, if the departed,
Being released from earth, should still retain
A sense of earthly pain;
It were a double grief if the true hearted,
Who loved us here, should on the further shore
Remember us no more.

Believing, in the midst of our afflictions,
That death is a beginning, not an end,
We cry to them, and send
Farewells, that better might be called predictions,
Being foreshadowings of the future, thrown
Into the vast Unknown.

Faith overleaps the confines of our reason,
And if by faith, as in old times was said,
Women received their dead
Raised up to life, then only for a season
Our partings are, nor shall we wait in vain
Until we meet again!

### SPEED AT WHICH WINGS ARE DRIVEN.

[From Frazer's Magazine.]

It has been estimated that the common fly moves its wings 330 times per second, *i. e.*, 19,800 times per minute, and that the butterfly moves its wings 9 times per second, or 540 times per minute. These movements represent an incredibly high speed, even at the roots of the wings; but the speed is enormously increased at the tips of the wings, from the fact that the tips rotate upon the roots as centres. The progressive increase in the speed of the wings, in proportion as the wings become larger, explains why the wings of bats and birds are not driven at the extravagant speed of insect wings. Thus, large gulls flap their wings much more slowly than small gulls; the relative size of the wings to the body being the same in both. This is a hopeful feature in construction of flying machines, as there can be no doubt that comparatively very slow movements will suffice for driving the long, powerful wings required to elevate and propel flying machines. Fifty or sixty pulsations of the wing per minute do not involve much wear and tear of the working parts, and I am strongly of opinion that artificial flight, if once achieved, will become a comparatively safe means of locomotion as far as the machinery required is concerned.

